# Minimally Invasive Transitional Full‐Mouth Adhesive Rehabilitation of a Patient With Amelogenesis Imperfecta Using Direct and Indirect Composite Restorations: A Case Report

**DOI:** 10.1155/crid/1565185

**Published:** 2026-10-14

**Authors:** Masoumeh Alavi, Masoud Sanei, Fatemehsadat Ahmadi

**Affiliations:** ^1^ Postgraduate Student in Restorative and Esthetic Dentistry Department of Restorative Dentistry School of Dentistry Tehran University of Medical Sciences (TUMS) Tehran Iran tums.ac.ir; ^2^ Specialist in Restorative and Esthetic Dentistry Department of Restorative Dentistry School of Dentistry Tehran University of Medical Sciences (TUMS) Tehran Iran tums.ac.ir

**Keywords:** amelogenesis imperfecta, case report, composite resins, digital diagnostic wax-up, indirect composite restoration

## Abstract

**Background:**

Amelogenesis imperfecta (AI) comprises a group of inherited conditions affecting enamel formation and mineralization, with significant esthetic, functional, and psychosocial consequences. Management of adolescents with hypocalcified AI remains challenging because of the compromised enamel structure, ongoing craniofacial growth, and need to preserve future restorative options. This report describes a staged direct and indirect composite approach in an adolescent with hypocalcified AI and concomitant Bartter syndrome, including its short‐term clinical performance and limitations.

**Case Presentation:**

A 15‐year‐old girl with hypocalcified AI and concomitant Bartter syndrome was referred with chief complaints of poor esthetics, hypersensitivity, and difficulty maintaining oral hygiene. Clinical and radiographic examinations revealed generalized enamel defects, discoloration, attrition, anterior open bite, and gingivitis. Given the patient′s age, systemic condition, and ongoing skeletal growth, a minimally invasive transitional rehabilitation was planned. Following digital diagnostic wax‐up and mock‐up evaluation, the maxillary arch was restored predominantly with indirect composite crowns, whereas the mandibular arch was restored with direct composite restorations without tooth preparation. Treatment was completed over 3 months.

**Results:**

At the 3‐ and 14‐month follow‐up examinations, the restorations were evaluated descriptively. They remained clinically functional and esthetically acceptable, except for debonding of the direct full‐coverage composite restoration on the retained mandibular right primary second molar, which was subsequently restored with a stainless‐steel crown. The patient reported subjective improvements in dental hypersensitivity, ability to maintain oral hygiene, smile appearance, and self‐confidence.

**Conclusion:**

Adhesive rehabilitation using a combination of indirect and direct composite restorations may provide a conservative transitional treatment option for adolescents with hypocalcified AI. In this case, the digital diagnostic wax‐up, mock‐up evaluation, and staged treatment facilitated communication and preoperative assessment of the proposed outcome while supporting preservation of the remaining tooth structure. Continued follow‐up is required, and the need for repair, replacement, or more definitive rehabilitation should be reassessed after further growth and maturation.

## 1. Introduction

Amelogenesis imperfecta (AI) is a group of inherited conditions characterized by alterations in enamel formation and mineralization, with considerable genetic, histological, and clinical heterogeneity [1]. AI may affect both the primary and permanent dentitions, and its clinical expression can vary substantially among and within affected individuals [2]. The reported prevalence of AI ranges from approximately 1 in 700 to 1 in 14,000, depending on the population studied [1].

Traditionally, AI has been clinically classified into hypoplastic (Type I), hypomaturation (Type II), hypocalcified (Type III), and hypomaturation‐hypoplastic AI with taurodontism (Type IV) [3]. Hypoplastic AI is primarily a quantitative enamel defect characterized by reduced enamel thickness, pitting, and grooves. In contrast, hypomaturation and hypocalcified AI are generally considered hypomineralized forms, in which enamel mineralization and structural integrity are impaired. Hypomineralized enamel typically exhibits lower radiodensity and inferior mechanical properties compared with sound enamel. Hypocalcified enamel may appear opaque and is particularly susceptible to discoloration and rapid wear, whereas hypomaturation enamel is often speckled and softer than sound enamel. Hypoplastic and hypomineralized enamel features may coexist in the same individual or even on different surfaces of the same tooth, contributing to the considerable clinical variability of AI [2].

AI may result in rapid enamel breakdown, tooth wear, dentin exposure, hypersensitivity or pain, compromised esthetics, and gingival inflammation, with potentially substantial psychosocial consequences and reduced oral health‐related quality of life [1]. Associated dental findings may include malocclusion, delayed eruption, abnormal tooth morphology, and attrition [2]. In more severely affected patients, enamel loss may lead to reduced proximal contacts, loss of occlusal vertical dimension, and reduced masticatory efficiency. Craniofacial characteristics, including anterior or posterior open bite, negative overjet, and altered vertical jaw relationships, have also been reported in patients with AI [4].

Restorative treatment planning for patients with AI should be individualized according to the patient′s age, phenotype and severity of enamel involvement, amount of remaining tooth structure, sensitivity, occlusal condition, esthetic demands, socioeconomic circumstances, and anticipated long‐term follow‐up. In children and adolescents, conservative approaches, including direct or indirect resin‐based restorations, are frequently considered because they preserve tooth structure and can be modified or repaired during ongoing tooth eruption and craniofacial growth [1, 3]. The restorative management of adolescents with AI remains challenging. Resin‐based restorations may provide an appropriate conservative or transitional solution in young patients, whereas indirect ceramic restorations may be considered in selected cases with more severe functional or esthetic impairment [1]. Although definitive prosthetic rehabilitation has traditionally been deferred until adulthood, clinical evidence suggests that ceramic crown therapy may be a feasible option for selected adolescents with severe AI [1, 5, 6]. The choice between resin‐based and ceramic restorative approaches should therefore be individualized, balancing the need for minimal intervention and future adaptability against the anticipated functional and esthetic demands of each case.

Because AI is rare, the available evidence regarding its restorative management is derived predominantly from case reports, case series, and observational clinical studies, limiting the certainty of treatment recommendations [1]. This case report describes the conservative esthetic and functional rehabilitation of an adolescent with AI and concomitant Bartter syndrome using direct and indirect composite restorations. The clinical rationale for the selected treatment, its limitations, and the need for continued follow‐up are discussed.

## 2. Clinical Report

A 15‐year‐old girl was referred by a general dentist to the Department of Restorative and Esthetic Dentistry at Tehran University of Medical Sciences with chief complaints of poor esthetics and dental hypersensitivity to thermal stimuli and tooth brushing. Written informed consent for treatment and publication of this case report, including identifiable clinical information and facial photographs with ocular masking, was obtained from the patient′s parents; the patient also provided assent for publication.

According to the patient′s history, both the primary and permanent teeth initially erupted with a relatively normal appearance. Approximately 1 year after eruption, however, progressive enamel changes became evident in both dentitions, including yellow‐brown discoloration, enamel fracture and breakdown, progressive occlusal wear, and dental hypersensitivity. At the time of examination, the dentition consisted predominantly of permanent teeth, with only one retained primary molar (Figure 1). There was no family history of similar dental abnormalities.

**Figure 1:**
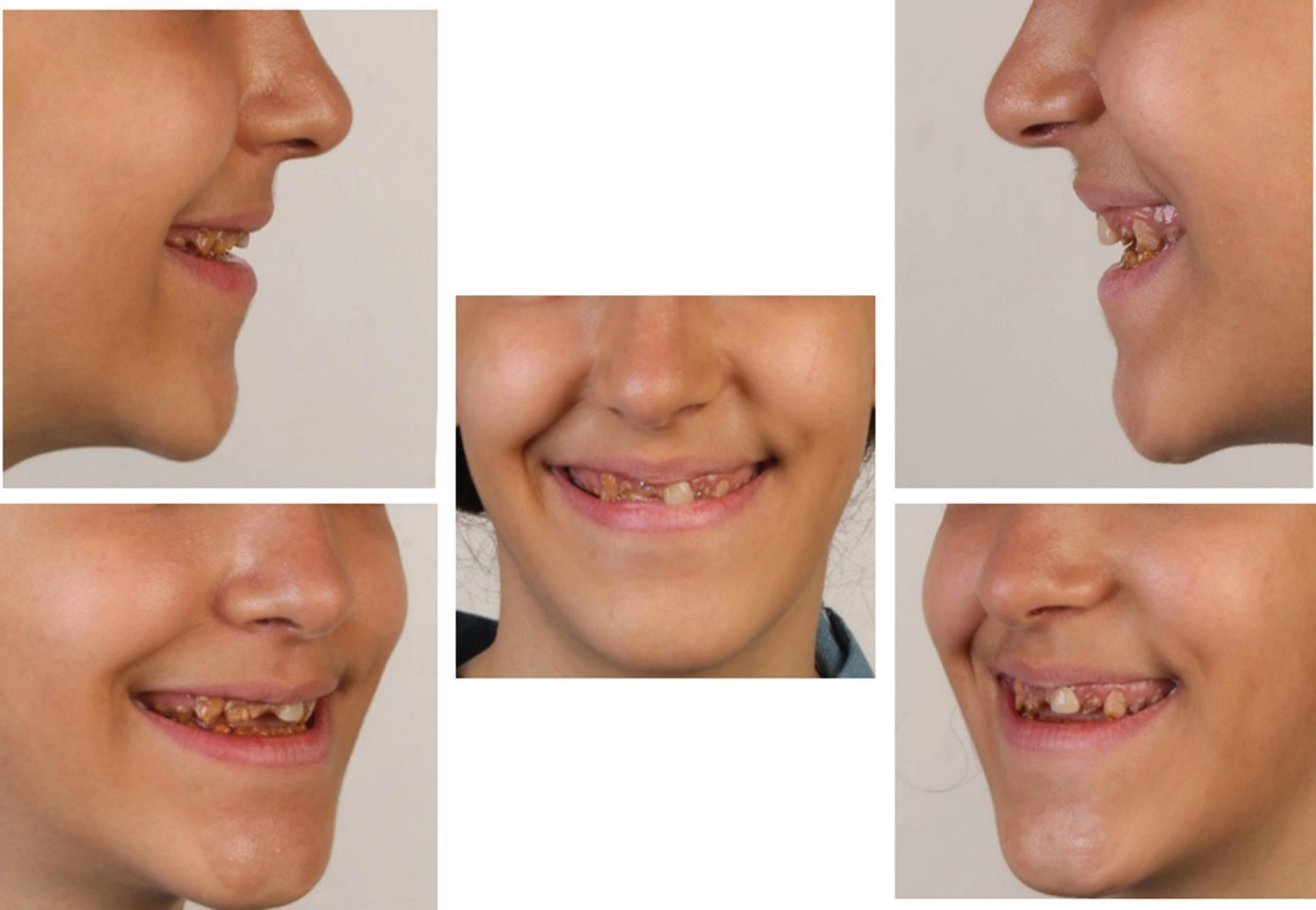
Profile, frontal, and three‐quarter smile view of the patient.

Radiographic examination revealed generalized enamel defects consistent with AI in the remaining teeth (Figure 2).

**Figure 2:**
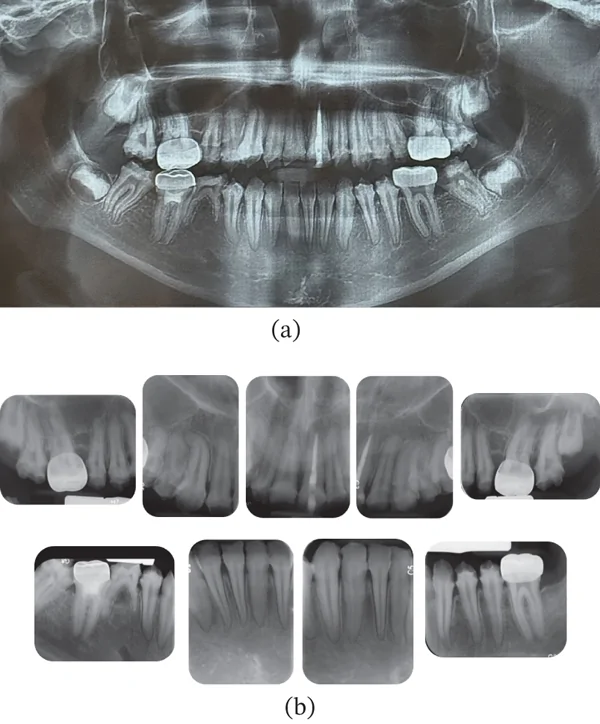
Preoperative radiographic view. (a) Panoramic view. (b) Periapical radiographs.

Prefabricated stainless‐steel crowns had been placed on the first permanent molars by a pediatric dentist at the age of 6 years to reduce the risk of further wear and caries.

The mandibular right second premolar (Tooth #45) was congenitally absent. The retained mandibular right primary second molar (Tooth #85) showed no evidence of root resorption, and its probing depths were within normal limits. The mandibular left second molar (Tooth #37) was also absent. One year earlier, the tooth had been extracted at a dental clinic because of associated pain. All four third molars were unerupted (Figure 2a).

The mandibular right first molar (Tooth #46) had previously undergone pulpotomy and was subsequently restored with a stainless‐steel crown.

The maxillary left central incisor (Tooth #21) had undergone root canal treatment at the age of 11 years and had been restored with a direct composite resin crown. Cold sensibility testing was performed on all erupted teeth except the root canal‐treated maxillary left central incisor (Tooth #21) and the teeth restored with stainless‐steel crowns (Teeth #16, #26, #36, and #46). All teeth subjected to cold testing responded within normal limits. Percussion testing and palpation of the associated periapical tissues were performed for all teeth, and no tenderness was detected.

On the periapical radiographs, slight widening of the periodontal ligament space and radiolucencies around the roots of some teeth were observed, particularly around the roots of the mandibular anterior teeth and in the furcation area of the retained primary molar (Figure 2b). Given the normal responses of the teeth subjected to cold sensibility testing, the absence of tenderness to percussion and palpation, and the lack of other associated clinical signs or symptoms, endodontic treatment was not indicated for these teeth.

At the 14‐month follow‐up, the previously observed periodontal ligament space widening and radiolucencies remained unchanged in size and radiographic appearance compared with the pretreatment findings, with no apparent progression. The patient remained asymptomatic, with no associated clinical signs.

Clinically, the exposed tooth surfaces exhibited yellow‐brown discoloration, rough and irregular enamel, chipping, and attrition (Figure 3). The patient reported difficulty maintaining adequate oral hygiene because of hypersensitivity. Clinical examination revealed substantial plaque accumulation and generalized gingivitis, particularly in the maxillary anterior sextant. Initial periodontal treatment, including scaling and oral hygiene instruction, was therefore provided. Early carious lesions were observed, especially in the maxillary second molars.

Occlusal examination revealed an Angle Class III relationship associated with an anterior open bite. Canine guidance was present on the left side, whereas group function was observed on the right side (Figure 3).

**Figure 3:**
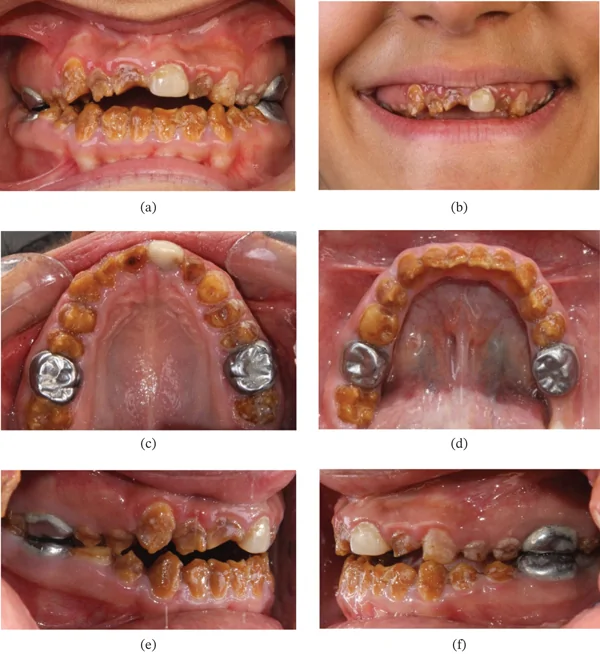
Preoperative view. (a) Smile view. (b) Retracted labial view. (c) Retracted occlusal view of maxillary arch. (d) Retracted occlusal view of mandibular arch. (e) Retracted right lateral view. (f) Retracted left lateral view.

The patient had a confirmed diagnosis of Bartter syndrome, established at 5 years of age by a pediatric nephrologist. She remained under continuous medical management with oral potassium chloride supplementation, indomethacin, and spironolactone. At the time of dental presentation, her serum electrolyte levels were within the normal laboratory reference ranges, with no clinically significant electrolyte imbalance reported.

Local anesthesia without a vasoconstrictor was selected as a patient‐specific precaution, considering the potential electrolyte and cardiovascular effects associated with Bartter syndrome, rather than because vasoconstrictors are universally contraindicated in this condition [7]. Given the patient′s reported muscular weakness and its potential association with hypokalemia in Bartter syndrome, prolonged treatment sessions were avoided as an additional patient‐specific precaution [8].

Based on the patient′s history and the clinical and radiographic findings, a clinical diagnosis of hypocalcified AI was made. Genetic testing was not performed; therefore, the molecular etiology of the enamel phenotype could not be established, and a syndromic genetic condition, including FAM20A‐associated enamel‐renal syndrome (ERS) [9], could not be definitively excluded.

Given the patient′s age and ongoing skeletal growth, the treatment objective was to provide a transitional restorative solution that would improve function and esthetics while preserving the possibility of future treatment modification. The need for more definitive rehabilitation would be reassessed after further growth and maturation.

## 3. Treatment Procedure

The preventive protocol included oral hygiene instruction, recommendations for the use of fluoride‐containing toothpaste, and dietary counseling aimed at reducing the risk of dental caries. These recommendations were reinforced at subsequent visits as part of ongoing preventive care. The patient also received professional supragingival scaling and polishing. At each subsequent visit, oral hygiene instructions were reinforced, and the gingival condition and degree of inflammation were clinically assessed to monitor improvement.

The treatment options were discussed with the patient and her parents. The final decision was to restore the maxillary teeth with indirect composite restorations and the mandibular teeth with direct composite restorations, which involved lower costs and required no tooth preparation.

### 3.1. Initial Bonding Evaluation

To obtain a preliminary clinical assessment of adhesive performance on the available bonding substrate, which consisted predominantly of exposed dentin with limited residual altered enamel, the maxillary second molars were restored with direct composite resin (Estelite Posterior, shade A3; Tokuyama, Japan). The restorations were monitored for 1 month and showed satisfactory retention and marginal integrity during this period (Figure 4). Based on these favorable short‐term findings, comprehensive restorative treatment was initiated.

**Figure 4:**
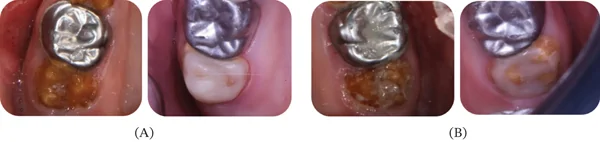
(A) Left maxillary second molar. (B) Right maxillary second molar.

### 3.2. Maxillary Arch Rehabilitation

To improve esthetic outcomes, reduce chairside restorative time, and encourage patient engagement at the beginning of treatment, indirect composite crowns were planned for Teeth #15–25 (FDI).

Minimal tooth preparation was performed without aggressive reduction or defined finish lines (Figure 5). Preparation consisted primarily of opening the proximal contacts to facilitate digital scanning. A retraction cord (Ultrapak 000, Ultradent, United States) was placed in the gingival sulcus, and a digital impression was obtained using an intraoral scanner (Helios 600, Eighteeth, China) (Figure 6A).

**Figure 5:**
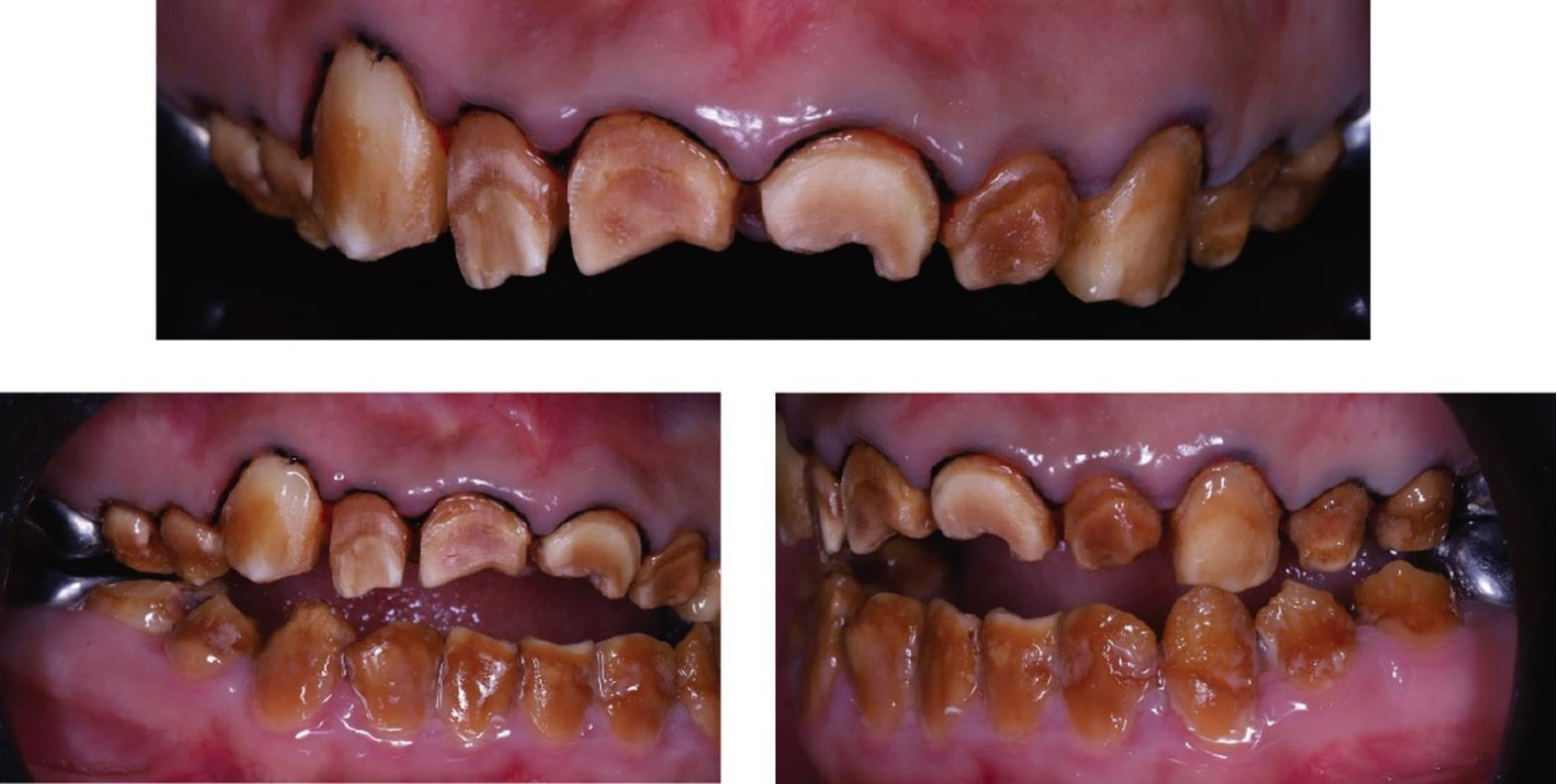
Initial preparation of the maxillary teeth for intraoral scanning and digital diagnostic wax‐up.

**Figure 6:**
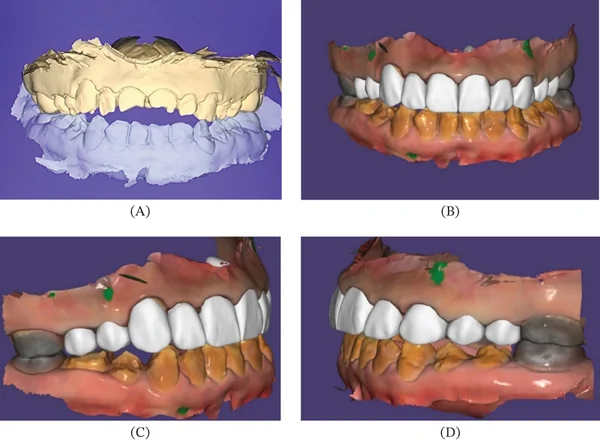
(A) Intraoral scan of maxillary and mandibular arches. (B) Frontal view of digital diagnostic wax‐up. (C) Right lateral view of digital diagnostic wax‐up. (D) Left lateral view of digital diagnostic wax‐up.

A digital diagnostic wax‐up was created using exocad software (exocad GmbH, Germany) based exclusively on the intraoral scan to plan the proposed tooth contours and minimize unnecessary tooth reduction. No facial photographs, facial scans, or lip‐related reference parameters were incorporated into the digital design. A model of the completed design was printed and used as a physical reference for fabrication of a transparent, 1‐mm‐thick, vacuum‐formed thermoplastic matrix (Forplast Roko). The matrix incorporated occlusal stops on the first permanent molars to provide a stable reference for accurate seating and vertical positioning. It was used to transfer the bis‐acryl material intraorally for mock‐up evaluation and provisionalization (Figures 6B–D and 7).

**Figure 7:**
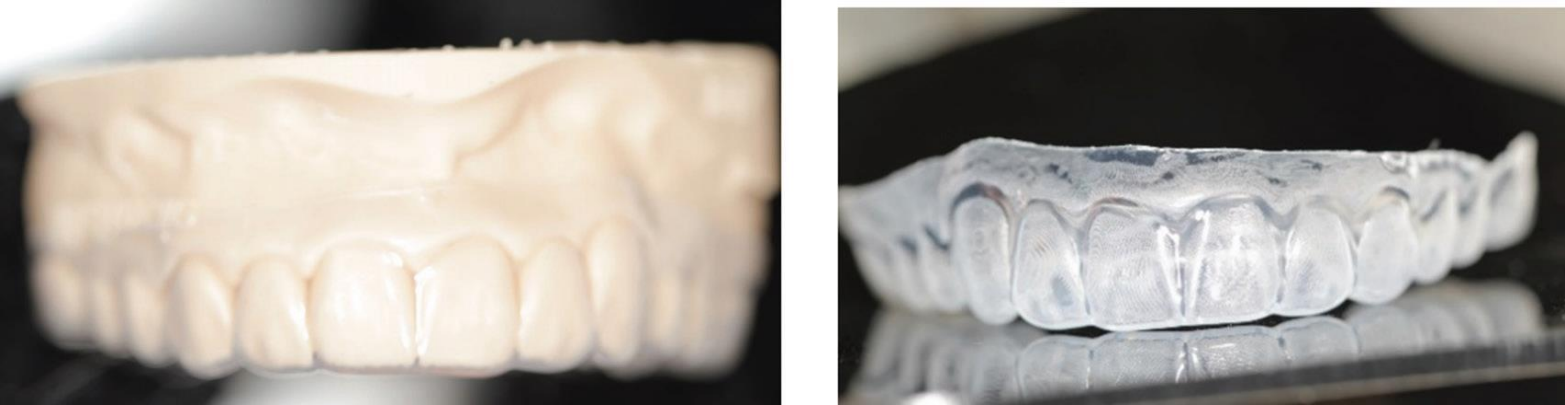
The 3D‐printed cast and a transparent thermoplastic vacuum‐formed tray fabricated for mock‐up and provisionalization.

The mock‐up was reviewed by the patient and clinically evaluated for esthetics, phonetics, lip support, and occlusal harmony (Figures 8 and 9). Following patient acceptance and clinical approval of the proposed design, selective additional tooth preparation was performed only in areas where residual discoloration remained clinically unacceptable (Figure 10). An updated intraoral scan was then obtained for fabrication of the definitive maxillary indirect composite restorations. A silicone putty index of the approved design was also fabricated and sent to the dental laboratory together with the updated digital scan to guide the planned restoration contours and composite thickness.

**Figure 8:**
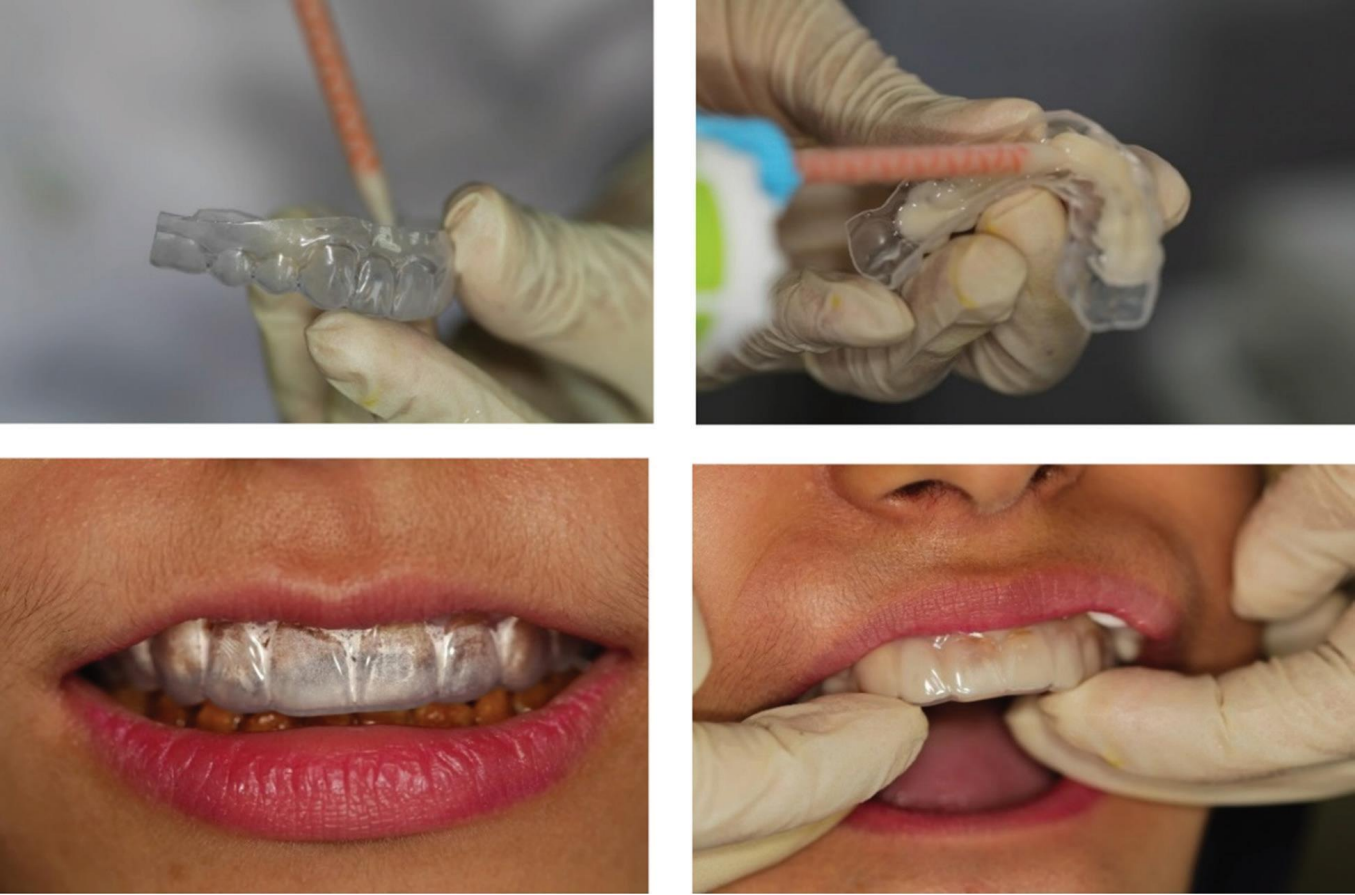
Temporary bis‐acryl was injected into the tray and then positioned intraorally.

**Figure 9:**
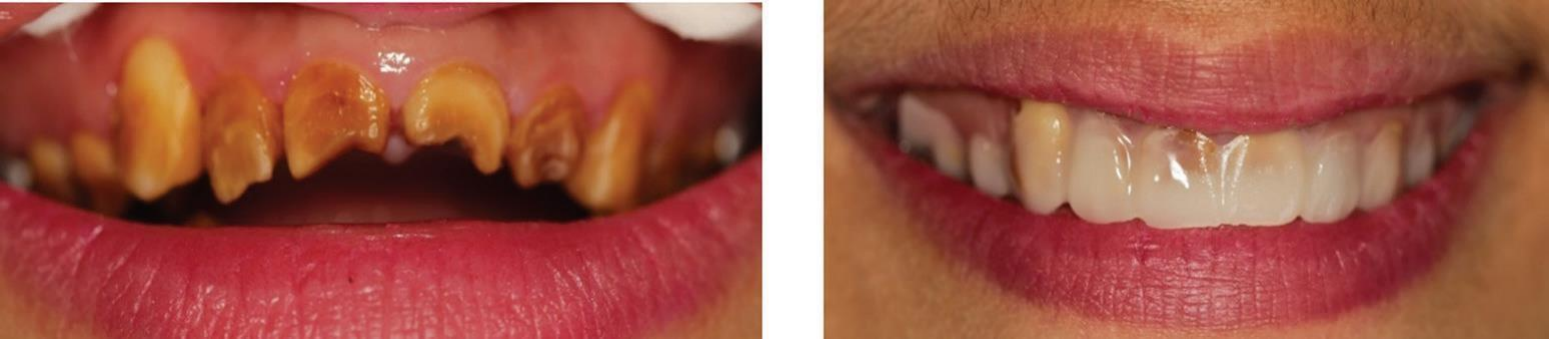
Based on the initial mock‐up, additional tooth reduction was required to mask the underlying discoloration.

**Figure 10:**
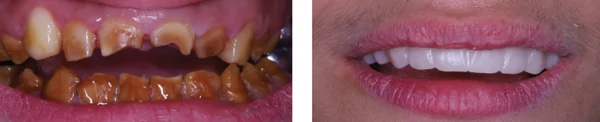
Preparation refinement and repeat mock‐up were performed prior to the final scan for fabrication of the indirect composite restorations.

For the definitive indirect composite restorations, the printed design and silicone putty index served as morphological references. The restorations were fabricated freehand by the laboratory technician on a 3D‐printed cast using a manual layer‐by‐layer technique. Because the selected composite material was not intended for CAD/CAM milling, the digital design was not transferred directly to the definitive restorations by milling.

The definitive restorations were fabricated using a supranano‐filled universal composite resin (Estelite Sigma Quick, Tokuyama, Japan; shades OPA2, OA2, and A2). A thin opaque layer (OPA2) was initially applied to mask the underlying discoloration. Dentin layering was then performed using shade OA2, followed by enamel layering with shade A2. Each composite layer was light‐cured separately after application. After completion of the layering procedure, the restorations were polymerized for 6 min under controlled conditions using a laboratory light‐curing unit (SCHÜTZ Spektra 2000 Fast).

Although the silicone index served as a morphological reference, the restorations returned from the laboratory showed slightly excessive facial contour and incisal length compared with the approved index. Minor extraoral adjustments were therefore performed to reproduce the planned contours and thickness, followed by finishing, polishing, and adhesive cementation (Figures 11 and 12).

**Figure 11:**
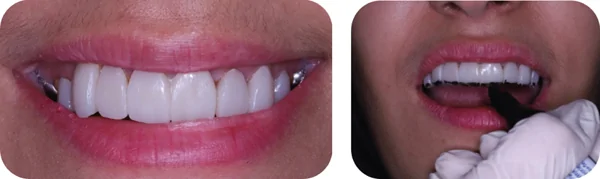
Refinement of the definitive composite restorations.

**Figure 12:**
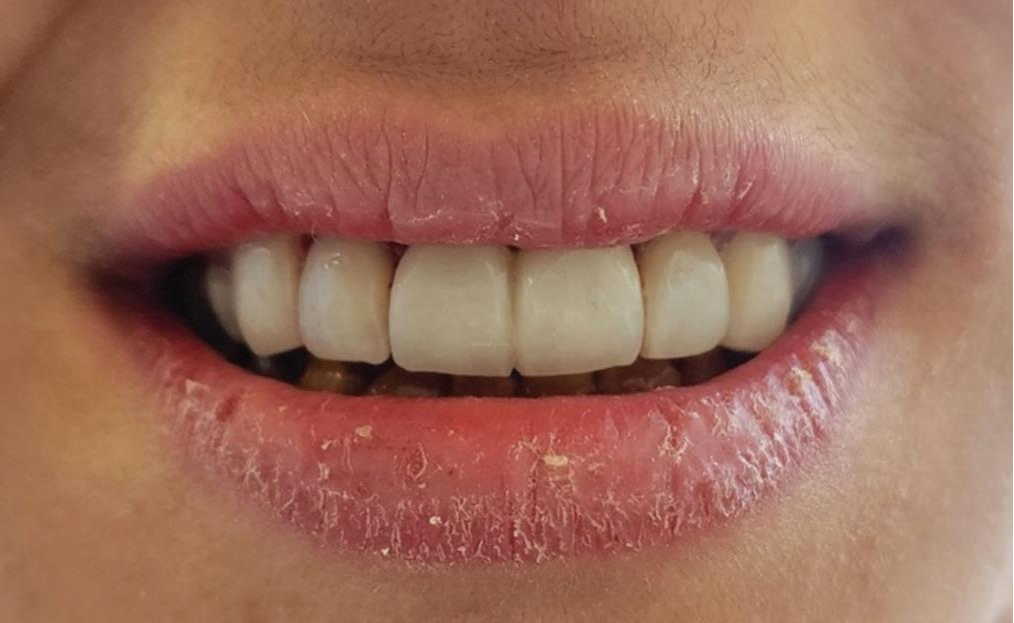
Clinical image of maxillary indirect composite restorations immediately after cementation.

### 3.3. Adhesive Protocol

The intaglio surfaces of the indirect composite restorations were subjected to airborne‐particle abrasion with 50‐*μ*m aluminum oxide (Al_2_O_3_) particles at 1.5 bar for 5 s, with the nozzle positioned approximately 10 mm from the restoration surface. The nozzle was moved continuously over the bonding surface to minimize localized overabrasion, particularly in thin areas of the restorations. Following airborne‐particle abrasion, 37% phosphoric acid was applied to the intaglio surfaces for 20 s to clean the treated surfaces and remove residual abrasive particles and debris. The restorations were then thoroughly rinsed with distilled water for 30 s and dried with oil‐free air. The treated surfaces were subsequently conditioned with Bis‐Silane Porcelain Primer (BISCO, Schaumburg, Illinois, United States) according to the manufacturer′s instructions.

Isolation was achieved using cotton rolls and high‐volume suction. An Ultrapak gingival retraction cord, size 000 (Ultradent Products, Inc., South Jordan, Utah, United States), was placed in the gingival sulcus during cementation of the indirect composite crowns.

The tooth surfaces were etched with 37% phosphoric acid for 30 s, rinsed, and gently dried. No deproteinizing agent, such as sodium hypochlorite, was applied before the adhesive procedure. All‐Bond Universal adhesive (BISCO, Schaumburg, Illinois, United States) was applied to the tooth surfaces and light‐cured for 20 s, according to the manufacturer′s instructions using an iLED Plus LED curing unit (Guilin Woodpecker Medical Instrument Co. Ltd., Guilin, China), with an irradiance of approximately 1000–1200 mW/cm^2^ and a wavelength range of 385–515 nm. The indirect composite crowns were then cemented using a light‐cure resin cement (Choice 2, shade B1; BISCO, Schaumburg, Illinois, United States). Because of the translucency of the indirect composite crowns, particularly in the incisal region, light curing was performed through the facial, palatal, and incisal surfaces to maximize light exposure and facilitate polymerization of the resin cement.

Using the same curing unit, the resin cement was light‐cured for 40 s from each aspect (facial, palatal, and incisal), resulting in a total exposure time of 120 s per restoration.

### 3.4. Mandibular Arch Rehabilitation

To reduce chair time and accommodate the patient′s systemic condition, the mandibular arch was restored over three appointments, progressing from the posterior to the anterior region (Figure 13).

**Figure 13:**
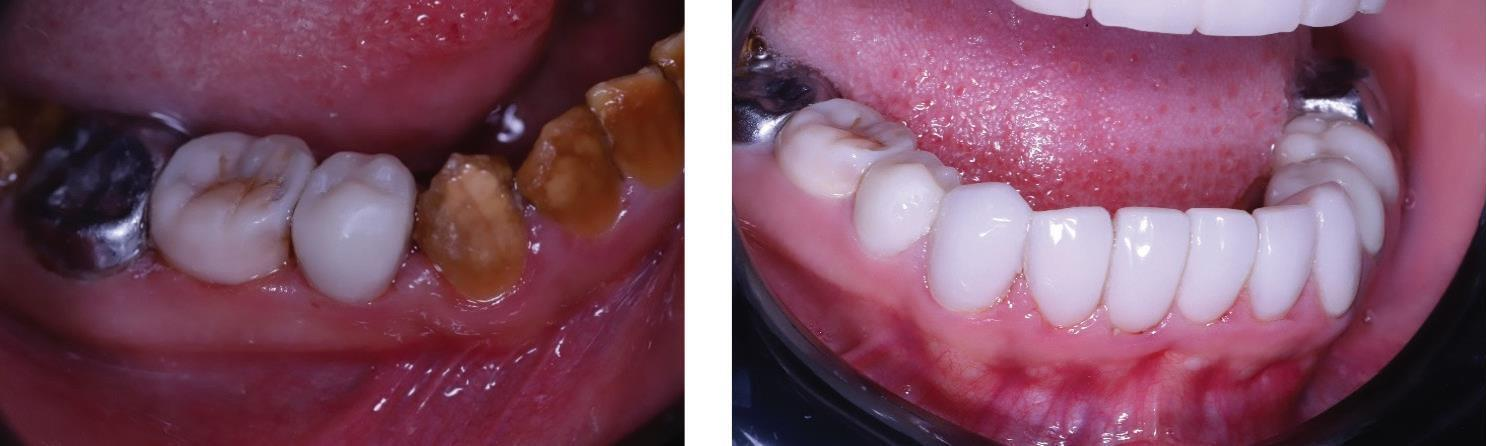
Reconstruction of the mandibular teeth from posterior to anterior.

The direct composite restorations were placed under isolation with cotton rolls and high‐volume suction. Matrix bands and wedges were used where required to restore the proximal contours. The tooth surfaces were prepared using the same adhesive protocol described for the indirect restorations: they were etched with 37% phosphoric acid for 30 s, rinsed, and gently dried. All‐Bond Universal was then applied and light‐cured according to the manufacturer′s instructions using the clinical curing unit described above. No sodium hypochlorite pretreatment was used. The same composite material and layering approach used for the maxillary crowns were used for the mandibular restorations. An opaque layer (OPA2) was initially applied to mask the underlying discoloration, followed by dentin layering with shade OA2 and enamel layering with shade A2. Each composite layer was light‐cured for 20 s using the same clinical curing unit. In some areas of the mandibular veneers, the OA2 or A2 layer was applied in a minimal thickness or omitted because of the limited restorative space resulting from the absence of tooth preparation.

The four mandibular anterior teeth (Teeth #31, #32, #41, and #42; FDI notation) received direct composite veneers limited to the facial surfaces. The other mandibular teeth included in the restorative treatment (Teeth #33, #34, #35, #43, #44, and #85) received direct full‐coverage composite restorations fabricated intraorally using Estelite Sigma Quick and the same layering and light‐curing protocol described above (Figure 13).

The pretreatment occlusal vertical dimension was assessed clinically and maintained throughout the rehabilitation. Restorative space was provided by minimal preparation of the maxillary teeth and the existing anterior open bite. The existing occlusal scheme, including left‐sided canine guidance and right‐sided group function, was preserved, and the occlusal contacts were verified after placement of the restorations.

### 3.5. Treatment Duration

The comprehensive transitional rehabilitation was completed over 3 months (Figure 14).

**Figure 14:**
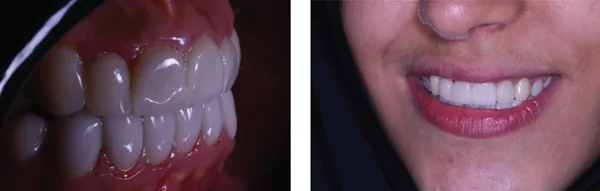
Clinical view of the composite restorations immediately after completion of the mandibular teeth treatment.

### 3.6. Recall

At the first recall appointment, 3 months after treatment completion, the restorations were evaluated descriptively for fracture and marginal adaptation, soft tissue response and postoperative sensitivity, and gloss and color match. They showed acceptable clinical performance and esthetic appearance, and the patient reported satisfaction with the treatment outcome (Figure 15). Minor chipping was observed on the occlusal surface of the direct full‐coverage composite restoration on the mandibular right primary second molar (Tooth #85). The area was polished to eliminate the sharp margins (Figure 15C).

**Figure 15:**
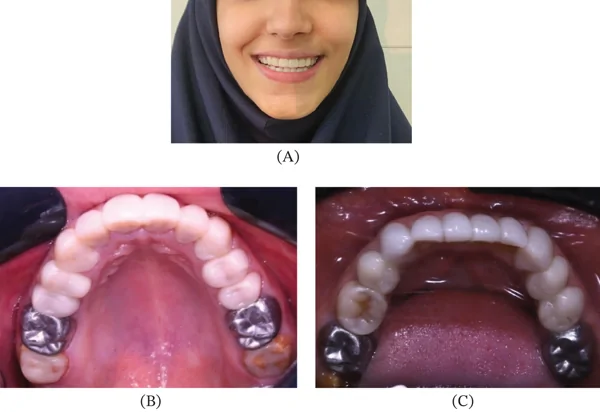
Clinical view of the composite restorations at the 3‐month follow‐up. (A) frontal view of the smile. (B) Retracted occlusal view of maxillary arch. (C) Retracted occlusal view of mandibular arch.

A second recall examination was performed 14 months after delivery of the restorations. All remaining composite restorations were intact and functional, with no evidence of chipping. The direct full‐coverage composite restoration on Tooth #85 had debonded 1 month earlier, approximately 13 months after placement, with only a small remnant remaining on the mesiolingual surface (Figure 16D). The patient was referred to the Department of Pediatric Dentistry at Tehran University of Medical Sciences, where the tooth was restored with a stainless‐steel crown (Figure 17).

**Figure 16:**
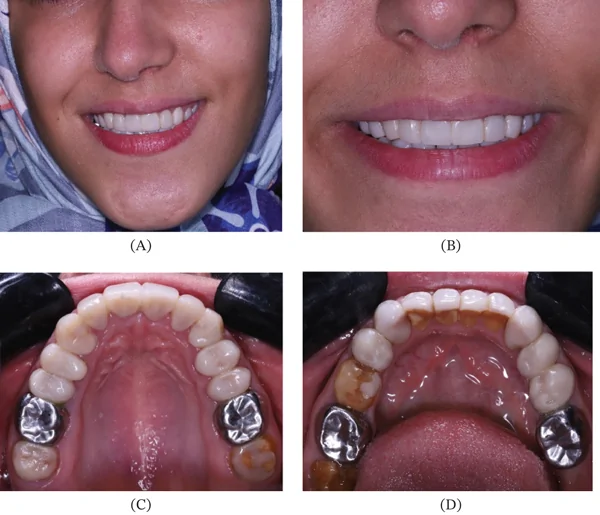
Clinical images of the composite restorations at the 14‐month follow‐up. (A) frontal view of the smile. (B) Smile view. (C) Retracted occlusal view of maxillary arch. (D) Retracted mandibular occlusal view showing debonded restoration of the right primary molar.

**Figure 17:**
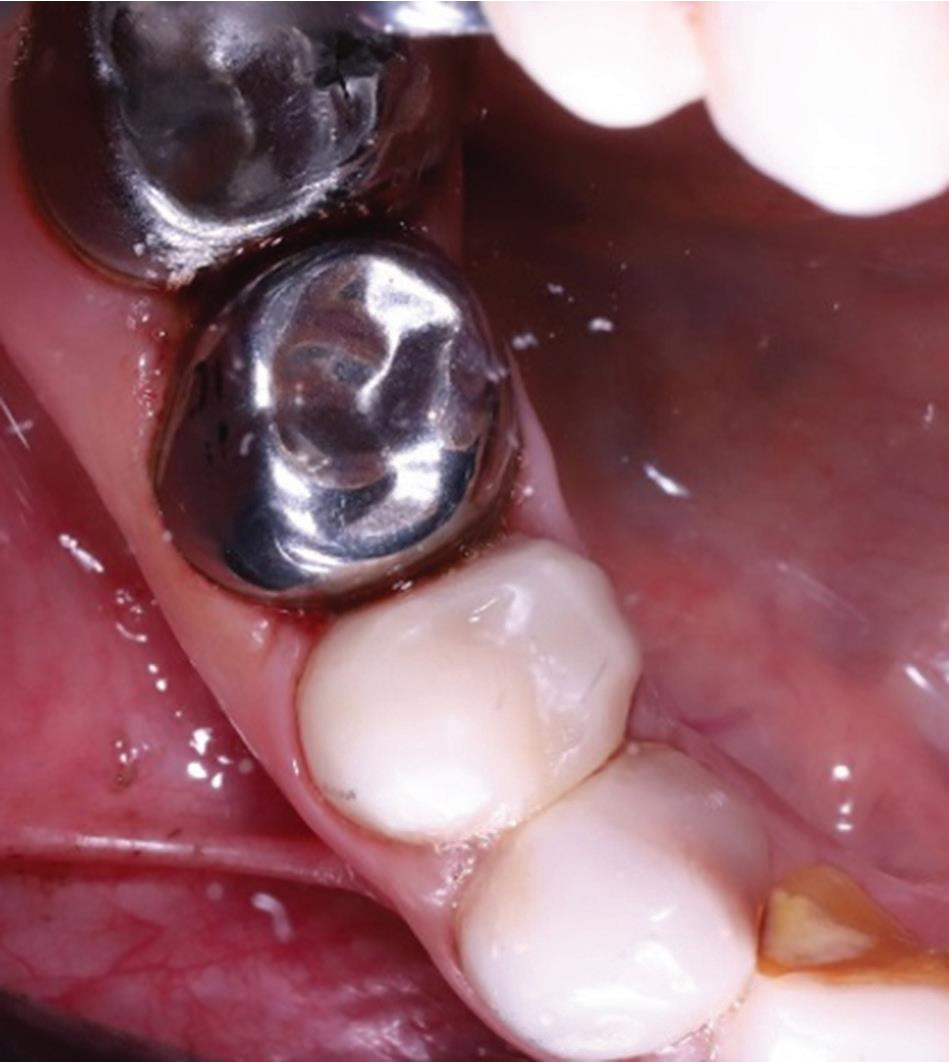
Placement of stainless steel crown (SSC) on the mandibular right primary molar.

The remaining restorations showed some marginal discoloration and loss of their original surface luster but remained clinically functional (Figure 16). The surrounding soft tissues showed a favorable clinical response. Oral hygiene instructions were reinforced, with particular emphasis on cleaning the cervical margins using the Bass brushing technique. The patient reported reduced dental hypersensitivity, an improved ability to maintain oral hygiene, greater satisfaction with her smile, and improved self‐confidence after treatment. These outcomes were based on the patient′s subjective perception.

At the 14‐month follow‐up, the previously observed slight widening of the periodontal ligament space and radiolucencies around some roots remained unchanged in size and radiographic appearance compared with the pretreatment findings, with no apparent progression (Figure 18). The patient remained asymptomatic, with no associated clinical signs.

**Figure 18:**
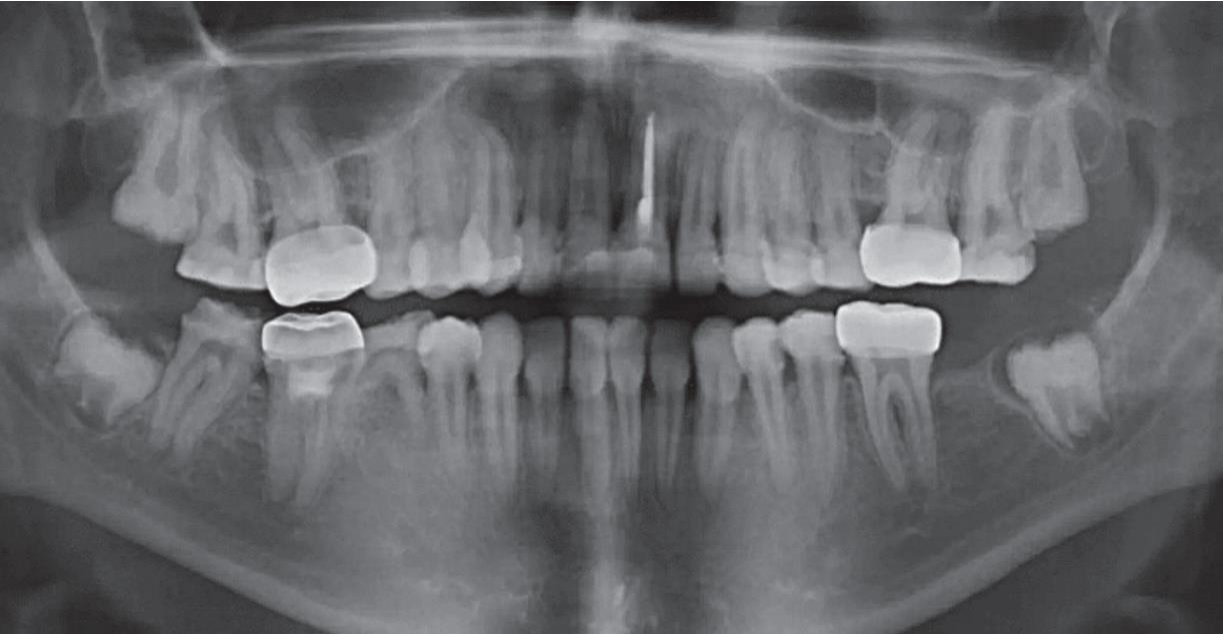
Panoramic view at the 14‐month follow‐up.

## 4. Discussion

This case report describes the minimally invasive esthetic and functional rehabilitation of a 15‐year‐old patient with AI and Bartter syndrome. A staged adhesive approach involving indirect composite crowns in the maxillary anterior region and direct composite restorations in the mandibular arch was selected according to the patient′s age, clinical condition, and the need to minimize tooth preparation. At the 14‐month follow‐up, the restorations remained clinically functional, except for debonding of the direct full‐coverage composite restoration placed on the mandibular right primary second molar. These findings should be interpreted cautiously because the follow‐up period is insufficient to determine the long‐term survival, wear behavior, color stability, or marginal performance of the restorations.

The relationship between the AI‐like enamel phenotype and Bartter syndrome deserves consideration in this case. AI‐like enamel abnormalities have been reported in patients with Bartter syndrome. Martelli‐Júnior et al. [10] identified typical AI features in two of eight patients with confirmed Bartter syndrome and suggested that renal tubulopathy may affect dental biomineralization. More recently, a pediatric case series of AI associated with renal abnormalities also included Bartter syndrome [11]. These reports raise the possibility that renal tubular dysfunction, altered renal mineral handling, and systemic mineral homeostasis may contribute to disturbances in dental biomineralization. The involvement of both the primary and permanent dentitions in this patient, together with progressive posteruptive enamel deterioration, may be consistent with this possibility. However, the available evidence is limited, and neither these clinical findings nor the coexistence of Bartter syndrome establishes a causal relationship with the enamel phenotype. A relatively normal appearance at eruption does not necessarily exclude an AI phenotype. In hypomaturation AI, enamel may have near‐normal volume but reduced structural integrity, resulting in rapid posteruptive enamel loss and fracture, whereas brown discoloration and early posteruptive enamel loss are characteristic of hypomineralized AI [9]. In this patient, both the primary and permanent teeth reportedly had a relatively normal appearance at eruption, followed approximately 1 year later by yellow‐brown discoloration, enamel fracture, progressive wear, and hypersensitivity. This clinical course is compatible with AI.

ERS, particularly that associated with biallelic FAM20A variants, was also considered in the differential diagnosis. ERS may present with AI, delayed tooth eruption, intrapulpal calcifications, and gingival hyperplasia [9]. There was no family history of similar dental abnormalities, and the complete ERS phenotype was not evident in this patient. However, genetic testing was not performed; therefore, a syndromic genetic etiology, including ERS, cannot be definitively excluded. The absence of molecular testing is acknowledged as a limitation of this report.

Management of individuals with AI has been described in temporary, transitional, and permanent phases, broadly corresponding to the primary or mixed dentition, adolescence, and adulthood, respectively. During the mixed and permanent dentition stages, the primary treatment objectives are to preserve tooth structure and pulpal vitality, maintain or restore the occlusal vertical dimension, and improve esthetics and function [12]. Treatment options for adolescents with AI range from conservative adhesive restorations to full‐coverage indirect rehabilitation. Their selection should be individualized according to the AI phenotype and severity, the amount and quality of residual tooth structure, functional and esthetic demands, growth status, and the feasibility of long‐term follow‐up [1, 12–16].

Stainless‐steel crowns are frequently used to protect severely affected primary and young permanent molars, particularly when preservation of arch space and occlusal vertical dimension is required [12, 17]. In this patient, prefabricated stainless‐steel crowns had been placed on the first permanent molars at the age of 6 years to reduce the risk of further enamel wear and caries.

During adolescence, conservative resin‐based restorations may be advantageous because they can often be placed with minimal tooth preparation, modified as eruption progresses, and repaired when necessary. They may also facilitate evaluation of the planned occlusion, tooth form, esthetics, and oral‐hygiene maintenance before more definitive rehabilitation is considered [1, 13–15].

A staged adhesive approach using direct and indirect composite restorations was selected to preserve the remaining tooth structure while improving esthetics, function, and sensitivity during adolescence. This minimally invasive approach was considered appropriate because further tooth eruption, gingival maturation, and craniofacial growth could still occur. However, resin‐composite restorations in AI may have limited longevity, particularly in severe hypomineralized, hypomaturation, or hypocalcified phenotypes associated with enamel breakdown and hypersensitivity [18, 19]. Their clinical performance may be influenced by disease severity, patient age, and the number of restored surfaces. Secondary caries, fracture, loss of restoration, and esthetic deterioration have been reported as common reasons for failure [1, 4]. In one clinical study, the 5‐year survival rate of composite resin restorations in patients with AI was approximately 50%, compared with 80% in matched controls. Restoration replacement was also 2.5 times more frequent in the AI group, with fracture or loss accounting for a substantial proportion of failures [18]. These findings should be applied cautiously to the present case because evidence derived from direct composite restorations may not be directly applicable to full‐coverage indirect composite restorations.

The quality of the bonding substrate is an important determinant of restorative prognosis in AI. Hypomineralized enamel may have reduced mineral content, increased protein content, and structural irregularities that interfere with etching, resin infiltration, resin‐tag formation, and micromechanical retention [12, 20]. Although some evidence regarding altered enamel properties has been derived from studies of molar‐incisor hypomineralization (MIH), these findings may provide indirect insight into adhesive challenges in other hypomineralized enamel conditions, including hypocalcified AI [20]. Direct composite restorations in hypocalcified AI have been associated with higher failure rates than those placed in other AI subtypes, probably because of the increased risk of enamel breakdown and reduced bonding performance of the affected enamel [4]. Failure may also occur cohesively within the defective enamel, at the dentinoenamel junction, or within dentin rather than at the adhesive interface alone [17]. Full‐coverage restorations may therefore be indicated for severely affected teeth with extensive enamel loss or an unfavorable bonding substrate [12]. For this reason, full‐coverage restorations were selected for the most severely affected teeth in the present case.

Indirect composite restorations may represent a useful conservative option for young patients with AI when preservation of the remaining tooth structure and future repairability are priorities. Extraoral fabrication can facilitate control of restoration contour, anatomy, proximal contacts, and surface finishing and polishing, while the relatively limited thickness of composite veneers may allow minimally invasive preparation in appropriately selected cases [21, 22]. Localized marginal defects, surface deterioration, or minor fractures may also be managed by intraoral composite repair rather than complete replacement of the restoration [21].

However, indirect composite restorations may involve higher costs and additional clinical or laboratory procedures compared with conventional direct composite restorations, and the available clinical evidence in patients with AI remains limited [21]. Clinical follow‐up of indirect composite veneers in non‐AI populations indicates that color mismatch, surface roughness, marginal discoloration, fracture, and debonding may occur over time, emphasizing the need for periodic maintenance and follow‐up [22].

Ceramic restorations represent an important alternative, particularly for patients with severe AI, as clinical studies have reported higher quality scores and greater longevity for ceramic crowns than for composite restorations [16, 19, 23]. Early ceramic crown therapy in adolescents with severe AI has shown encouraging medium‐ and long‐term outcomes, with reported crown survival rates of 99.6% after a mean follow‐up of 5.5 years and 97.8% after a mean follow‐up of 13.4 years. Continued eruption during adolescence may result in exposure of crown margins; however, in the cited studies, exposed margins of all‐ceramic crowns were generally not associated with clinically disturbing discoloration or patient‐reported esthetic concerns [5, 6]. Early prosthetic rehabilitation may also reduce the need for repeated replacement of composite restorations and has been associated with lower cumulative treatment costs when initiated during early adolescence rather than postponed until adulthood [5, 24].

Nevertheless, ceramic rehabilitation in young patients should be individualized rather than considered mandatory. Relatively large pulp chambers, short clinical crowns, continued craniofacial growth, further eruption, and potential changes in gingival margin position may complicate tooth preparation and alter marginal relationships over time [12, 13, 15, 16]. Extensive tooth preparation may also increase the risk of pulpal exposure, whereas the compromised enamel substrate may adversely affect adhesive procedures and long‐term performance [25]. When preservation of tooth structure, future modification, and intraoral repairability are priorities, indirect composite restorations may therefore provide a reasonable transitional esthetic and functional alternative during adolescence [21, 22, 25].

In this patient, indirect composite restorations were selected for the maxillary anterior teeth to facilitate extraoral control of tooth form, contour, anatomy, and surface finishing while limiting tooth preparation. This approach was considered appropriate given the patient′s age, ongoing development, and underlying medical condition because it reduced the extent of chairside restorative procedures. Direct composite restorations were used in the mandibular arch to provide a more conservative and readily modifiable treatment. The patient′s favorable acceptance of the maxillary rehabilitation also appeared to increase her willingness to proceed with the mandibular restorative treatment.

Accordingly, the current composite restorations should not be regarded as unequivocally permanent. They represent a conservative transitional rehabilitation requiring periodic clinical review of wear, marginal integrity, color stability, periodontal health, and functional performance. After further craniofacial growth and maturation, the need for repair, replacement, or more definitive indirect restorative options, including ceramic rehabilitation, should be reassessed according to the patient′s clinical, functional, esthetic, and biological conditions.

Prefabricated composite veneers and dedicated indirect composite systems have been proposed as conservative restorative options for young patients with AI. Controlled extraoral polymerization may improve selected material properties, including color stability, surface smoothness, and microhardness. However, the supporting evidence is predominantly laboratory‐based, and comparative clinical data remain limited [21] These potential advantages should be applied cautiously to the present case because the maxillary restorations were fabricated from a direct restorative composite material (Estelite Sigma Quick) using an indirect technique rather than from a dedicated prefabricated or indirect composite system. The clinical outcome should therefore be interpreted in relation to the material and technique used and should not be directly extrapolated to dedicated indirect composite systems.

A digital diagnostic wax‐up and intraoral mock‐up were used to communicate the proposed treatment outcome and evaluate esthetics, phonetics, lip support, and occlusal harmony before irreversible procedures were performed [26–28]. This workflow supported conservative treatment planning by allowing additional preparation only in areas where residual discoloration remained clinically unacceptable.

The bonding substrate in this case was mixed but consisted predominantly of exposed dentin, with limited residual enamel remaining mainly in the cervical regions. This distribution was important in selecting and interpreting the adhesive protocol. In advanced hypocalcified AI, substantial enamel loss and wear may expose the underlying dentin; therefore, the bonding strategy should be based on the actual substrate rather than the diagnosis alone.

Limited in vitro evidence suggests that hypocalcified AI may adversely affect bonding to both enamel and dentin. Faria‐e‐Silva et al. [29] reported lower bond strengths to AI‐affected tissues than to sound tooth tissues and found higher bond strength to dentin than to enamel in affected teeth. They also reported a positive association between enamel hardness and bond strength, supporting the view that poor mineralization and structural disorganization of the affected enamel might contribute to reduced adhesive performance. Although these findings do not establish that dentin exposure is universally advantageous, they suggest that retaining severely disorganized hypocalcified enamel may not provide a more favorable bonding substrate in severely affected teeth.

Phosphoric acid etching was performed before application of All‐Bond Universal rather than relying solely on the self‐etching capacity of the universal adhesive. A recent systematic review found low‐certainty evidence suggesting that an etch‐and‐rinse strategy may improve bonding to AI‐affected enamel and dentin compared with self‐etch approaches [30]. This approach was considered reasonable in the present case because of the structurally altered substrate and the predominance of dentin bonding. However, the optimal phosphoric acid etching duration for AI‐affected dentin remains uncertain. Hiraishi et al. [31] found no statistically significant improvement in bond strength when the etching time for primary dentin affected by AI was increased from 15 to 30 s. Consistent with this finding, the evidence supporting 30‐s etching of AI‐affected dentin has been characterized as being of very low certainty [30]. The 30‐s etching protocol used in this case should therefore not be interpreted as definitively superior.

Sodium hypochlorite deproteinization has been proposed to improve bonding to hypocalcified enamel by removing organic material that may interfere with resin infiltration. Although Şaroğlu et al. [32] reported increased shear bond strength to hypocalcified AI enamel after treatment with 5% NaOCl following phosphoric acid conditioning, this finding has not been consistently reproduced. In permanent teeth affected by hypocalcified AI, Faria‐e‐Silva et al. [29] found no significant effect of 5% NaOCl on bond strength to either enamel or dentin. A systematic review characterized the evidence for NaOCl deproteinization in AI as very limited and inconsistent, with any potential benefit appearing more relevant to enamel than to dentin [30]. Because the bonding substrate in the present case was predominantly dentin, sodium hypochlorite pretreatment was not used. The adhesive protocol was thus selected according to the actual substrate and the limited, low‐certainty evidence available for AI‐affected tissues.

For cementation of the relatively thin and translucent full‐coverage indirect composite crowns, a light‐cure resin cement was selected because sufficient light transmission was anticipated, particularly in the incisal region. Light curing was performed from the facial, palatal, and incisal aspects with extended exposure; however, adequate polymerization in less accessible areas could not be directly verified. A light‐cure resin cement was also considered potentially advantageous from an esthetic perspective because the absence of a chemical‐curing component may reduce the risk of color change over time compared with some dual‐cure resin cements [33]. Nevertheless, this choice represents a limitation of the present case because a dual‐cure resin cement might provide more predictable polymerization in areas of increased restoration thickness or reduced translucency.

The absence of periodontal correction for gingival asymmetries should also be acknowledged as a limitation of the treatment. This decision was made in view of the patient′s age and incomplete skeletal growth; therefore, restorative rehabilitation was planned within the existing gingival architecture. Future periodontal re‐evaluation should be based on gingival maturation, esthetic concerns, and the stability of the restorative outcome.

Regular follow‐up is important for monitoring gingival and periodontal health, reinforcing oral hygiene, and reducing the risk of secondary caries and periodontal deterioration [25]. At the 3‐month follow‐up, no restoration failure was observed. At the 14‐month recall, all restorations remained clinically functional except for the direct full‐coverage composite restoration on the mandibular right primary second molar, which had almost completely debonded, with a small remnant remaining on the mesiolingual surface. The reason for this failure cannot be determined from a single case. Potential but unverified contributing factors include the different bonding characteristics of primary tooth tissues, increased functional loading associated with the pre‐existing right‐sided group function, and limited occlusal thickness of the composite crown in a patient with anterior open bite and concentrated posterior occlusal contacts. The tooth was subsequently restored with a stainless‐steel crown.

This report has several limitations. As a single case report, it cannot establish the effectiveness, predictability, or long‐term survival of the described restorative approach, and its findings cannot be generalized to other patients with AI or AI‐like enamel abnormalities. The 14‐month follow‐up is insufficient to assess long‐term clinical performance, including wear, color stability, marginal integrity, debonding, fracture, periodontal response, and the need for repair or replacement. Genetic testing was not performed; therefore, the molecular etiology of the AI phenotype could not be established, and a syndromic genetic condition, including, could not be definitively excluded. No histological or quantitative assessment of enamel mineralization was available, and the proposed hypocalcified AI‐like phenotype was based on clinical and radiographic findings. Finally, longitudinal patient‐specific data on electrolyte status, systemic medication use, salivary parameters, and their potential influence on the oral environment and long‐term restorative prognosis were unavailable. No causal relationship between Bartter syndrome and either the enamel phenotype or restorative outcome can therefore be inferred from this case.

Despite these limitations, this case illustrates a conservative, staged restorative option for improving esthetics and function in an adolescent with AI and Bartter syndrome while preserving the possibility of future treatment modification.

## 5. Conclusion

The management of hypocalcified AI in adolescents is clinically challenging because of the defective enamel structure, compromised bonding substrate, ongoing craniofacial growth, and psychosocial concerns. Within the limitations of this case report and its 14‐month follow‐up, minimally invasive transitional rehabilitation with indirect and direct composite restorations provided acceptable esthetic and functional outcomes while preserving the remaining tooth structure. The restorations remained clinically functional except for debonding of one direct full‐coverage composite restoration. The patient also reported improvements in oral hygiene, hypersensitivity, and self‐confidence. Digital diagnostic wax‐up and mock‐up evaluation facilitated treatment planning, communication, and preoperative assessment of the proposed outcome. Indirect composite restorations may offer a conservative and repairable transitional option for young patients with AI when preservation of tooth structure and future treatment adaptability are priorities. Continued clinical follow‐up and reassessment after further growth and maturation remain necessary.

## Author Contributions

Masoumeh Alavi: writing—original draft, writing—review and editing, investigation, supervision, data curation, and validation. Fatemehsadat Ahmadi: conceptualization, methodology, project administration, visualization, data curation, resources, writing—original draft, and writing—review and editing.

## Funding

No funding was received for this manuscript.

## Disclosure

All authors have read and approved the final version of the manuscript. The corresponding author had full access to all of the data in this study and takes complete responsibility for the integrity of the data and the accuracy of the data analysis.

## Ethics Statement

This manuscript reports a single clinical case and does not involve experimental intervention or research‐specific procedures. No ethical committee approval was obtained for the preparation and publication of this case report. Written informed consent for publication of the case report and the accompanying identifiable clinical photographs was obtained from both the patient and her parents.

## Conflicts of Interest

The authors declare no conflicts of interest.

## Patient Perspective

The patient reported that dental hypersensitivity before treatment had made oral hygiene difficult and that she had been dissatisfied with the appearance of her teeth. Following treatment, she perceived a reduction in dental hypersensitivity and found it easier to maintain oral hygiene. She was satisfied with the appearance of her smile and reported an improvement in her self‐confidence. These outcomes reflect the patient′s subjective perspective and were not assessed using standardized questionnaires.

## Supporting information


**Supporting Information** Additional supporting information can be found online in the Supporting Information section. File S1: Figure files and figure citation.

## Data Availability

The data that support the findings of this study are available from the corresponding author upon reasonable request.
