# Supplementary material for: Minimally Invasive Transitional Full‐Mouth Adhesive Rehabilitation of a Patient With Amelogenesis Imperfecta Using Direct and Indirect Composite Restorations: A Case Report
Source: Case Rep Dent. 2026 Oct 14;2026:1565185. doi: 10.1155/crid/1565185 (PMC13662737; doi:10.1155/crid/1565185)
Supplement: Supplementary file 1 [file CRID-2026-1565185-s001.docx]

Dear Editorial Office,

Thank you for your message.

We have uploaded all 18 figures as separate editable Word files, as requested. In addition, we have carefully checked the manuscript and highlighted the citations for the corresponding figures within the main text to ensure that all figure references are clearly identifiable.

Please let us know if any further information or modifications are required.

Kind regards,
Dr. Fatemesadat Ahmadi
